# Internet of Things Long-Range-Wide-Area-Network-Based Wireless Sensors Network for Underground Mine Monitoring: Planning an Efficient, Safe, and Sustainable Labor Environment

**DOI:** 10.3390/s24216971

**Published:** 2024-10-30

**Authors:** Carlos Cacciuttolo, Edison Atencio, Seyedmilad Komarizadehasl, Jose Antonio Lozano-Galant

**Affiliations:** 1 Department of Civil Works and Geology, Catholic University of Temuco, Temuco 4780000, Chile; 2 Department of Civil Engineering, Universidad de Castilla-La Mancha, Av. Camilo Jose Cela s/n, 13071 Ciudad Real, Spainjoseantonio.lozano@uclm.es (J.A.L.-G.); 3 School of Civil Engineering, Pontificia Universidad Católica de Valparaíso, Av. Brasil 2147, Valparaíso 2340000, Chile; 4 Department of Civil and Environment Engineering, Universitat Politècnica de Catalunya, BarcelonaTech, C/Jordi Girona 1-3, 08034 Barcelona, Spain; milad.komary@upc.edu

**Keywords:** underground mine, digital mine, wireless sensors network, Internet of Things (IoT), LoRaWAN, productivity, safety, sustainability

## Abstract

Underground mines are considered one of the riskiest facilities for human activities due to numerous accidents and geotechnical failures recorded worldwide over the last century, which have resulted in unsafe labor conditions, poor health outcomes, injuries, and fatalities. One significant cause of these accidents is the inadequate or nonexistent capacity for the real-time monitoring of safety conditions in underground mines. In this context, new emerging technologies linked to the Industry 4.0 paradigm, such as sensors, the Internet of Things (IoT), and LoRaWAN (Long Range Wide Area Network) wireless connectivity, are being implemented for planning the efficient, safe, and sustainable performance of underground mine labor environments. This paper studies the implementation of an ecosystem composed of IoT sensors and LoRa wireless connectivity in a data-acquisition system, which eliminates the need for expensive cabling and manual monitoring in mining operations. Laying cables in an underground mine necessitates cable support and protection against issues, such as machinery operations, vehicle movements, mine operator activities, and groundwater intrusion. As the underground mine expands, additional sensors typically require costly cable installations unless wireless connectivity is employed. The results of this review indicate that an IoT LoRaWAN-based wireless sensor network (WSN) provides real-time data under complex conditions, effectively transmitting data through physical barriers. This network presents an attractive low-cost solution with reliable, simple, scalable, secure, and competitive characteristics compared to cable installations and manually collected readings, which are more sporadic and prone to human error. Reliable data on the behavior of the underground mine enhances productivity by improving key performance indicators (KPIs), minimizing accident risks, and promoting sustainable environmental conditions for mine operators. Finally, the adoption of IoT sensors and LoRaWAN wireless connectivity technologies provides information of the underground mine in real-time, which supports better decisions by the mining industry managers, by ensuring compliance with safety regulations, improving the productive performance, and fostering a roadmap towards more environmentally friendly labor conditions.

## 1. Introduction

In underground mining, there has always been a challenge, safety, where the risk of accidents is constant. In this sense, a brief introduction is presented below to understand the research problem and thus visualize alternative solutions for the wireless monitoring systems with sensors that could improve some aspects of complex working environmental conditions.

### 1.1. Operational Challenges of Underground Mines

Underground mining is a complex and high-risk activity due to its dynamic nature and development in conditions usually inhospitable to humans [1]. Underground mine activities consist of extracting and hauling material (ore recovery and mine waste extraction) through tunnels, galleries, and ramps that reach the surface. Material extraction must be based on safety and the economy while providing adequate roof and floor support at the production fronts to preserve the surface from subsidence [2].

**Figure 1:**
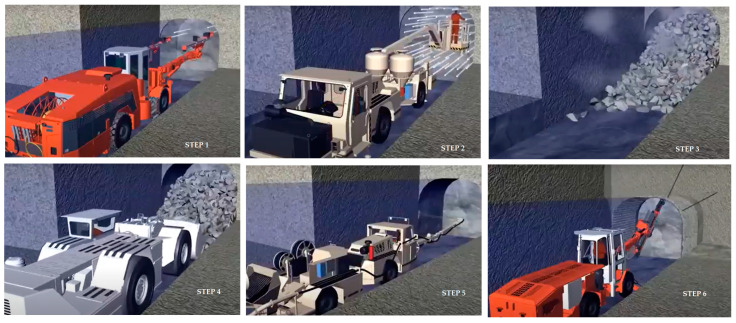
Main activities developed in a typical medium- or high-industrial-scale underground mine. Step 1: drilling; step 2: charging; step 3: blasting/ventilation; step 4: loading/ore hauling; step 5: concrete spraying; and step 6: bolting.

For example, each underground mine is unique considering the exploitation method selected to extract the minerals, where the main methods considered worldwide are as follows: (i) chambers and pillars, (ii) sub-level stopping; (iii) sub-level caving, (iv) block caving, and (v) subsidence, among others. Thus, each of the exploitation methods considers various activities, such as, for example, among the most important, (i) drilling, (ii) blasting, (iii) ventilation (entry of fresh air and extraction of toxic gases and dust), (iv) loading of ore, (v) hauling or transporting ore and mine waste rock material, (vi) removal of groundwater, (vii) providing services, electricity input, and radio communication, (viii) and support installation (bolts anchoring, shotcrete, and wire mesh), among others [2,3,4] (See Figure 1). 

On the other hand, this is how mining personnel, in addition to different vehicles and machinery, must perform different jobs. Some of the mechanical equipment considered are the following: (i) scoop tram (loading ore or mine waste rock), (ii) jumbo drill (drilling activities), (iii) dump truck (hauling ore or mine waste rock), and (iv) pickup trucks (personnel transport), among others. In this sense, it is important to mention that the personnel who work within the underground mine are not only workers from the mine itself but also other professionals from various disciplines to perform different tasks, among the following: (i) geologists, (ii) risk prevention engineer, (iii) company in charge of exploiting the underground mine, (iv) specialist technicians, suppliers/vendors and engineers from engineering/consultancy companies, and (v) mining authority inspectors, among others [5].

Underground mining must face numerous risks that endanger the human lives of its workers. These include exposure to the following: (i) electric shocks, (ii) noise, (iii) vibrations, (iv) extreme temperatures, (v) gases and vapors, (vi) acid aerosols, (vii) dust, (viii) viruses, (ix) bacteria, (x) fungi, (xi) parasites, (xii) rock explosions or rock bursts, (xiii) block falls, (xiv) highly faulted roofs, (xv) collapses, (xvi) floods, (xvii) collisions, (xviii) run over by machinery, (xix) fires, (xx) falling rocks due to lack of support, (xxi) presence of a large number of open galleries, (xxii) remnants of explosives left at the work face, (xxiii) low oxygen level inside the mine, (xxiv) excessive presence of carbon monoxide, and (xxv) falls into shafts, among others [6,7].

### 1.2. Why Is Underground Mine Monitoring Needed? A Growing Safety Issue

Nowadays, a real-time monitoring system with functions 24 h, 365 days per year, is rare in underground mines, which is surprising considering the risks that these infrastructures pose for miners. Considering the experience of mining engineering, civil engineering, and environmental engineering, the accident rate of underground mines over the last 2 decades is estimated to have an increasing trend [8].

In this context, the need for regular underground mine monitoring is demanded by the need to register for changing factors of safety (FoSs) throughout the lifetime of the mining projects [9]. FoSs are a key performance indicator in an underground mine environment because they have a direct relationship with the risks and, in this sense, are relevant to monitoring that evolution to maintain these risks as low as reasonably practicable (ALARP). In engineering, an FoS expresses how much stronger a human infrastructure system is than it needs to be for an intended external load [10]. Also called a safety factor, the FoS is a critical measure; if a multidisciplinary engineering team gets it wrong, an accident or disaster could ensue. The fact that accidents or disasters have occurred shows that underground mines’ FoS calculation methodologies are still evolving within the mining industry. A key issue in underground mines is that the engineering parameters used to calculate the FoS can change over time, so an underground mine that is deemed stable upon construction may become unstable later. These changes can affect not only the FoS itself but also the probability of accident or geotechnical failure [1].

Given the risks of accidents or geotechnical failures that are present in underground mines, the following gaps are fascinating to discuss and analyze: why the level of monitoring is still a challenge. In this sense, the costs of monitoring programs act as an obstacle, and it is certainly true that for several underground mines in remote areas, the budget required for regular surveys by qualified/expert professionals would be significant. However, any underground mining project or development in monitoring needs to be weighed against the potential economic and reputation loss linked with an underground accident or geotechnical failure—and this is considerable [11]. On the other hand, the growth of the mining technology market is driving greater attention to people’s safety and health, encouraging the adoption of IoT solutions, and increasing the implementation of autonomous equipment. However, the lack of highly qualified labor and the accessibility of an efficient infrastructure limit technology market growth [12].

According to these threats to efficient production, safety, and sustainability, investment in sensor monitoring appears to represent a cost-effective form of insurance. Furthermore, recent developments in technology mean that sensor monitoring no longer requires on-site or in-field human surveys, which means that most of the sensor monitoring can be performed remotely [13]. The key is to use smart sensors and the IoT to regularly monitor environmental conditions in underground mines and then send the monitoring data off for remote analysis outside of the mining infrastructure [14]. 

Nowadays, it is easy to install sensors for a wide range of variables to be monitored in underground mine landscapes [15], including the following:

- Tunnels and ramp deformation at various depths (prevent accidents due to rock bursting).
- Movement across surface cracks in tunnels and ramps.
- Air quality in tunnels (prevent inhalation of dust and harmful gases).
- Horizontal and vertical accelerations induced by seismic activity.
- Groundwater quantity measurement in tunnels.
- Groundwater quality measurement in tunnels.
- Groundwater level measurement in reservoirs.
- Global positioning system for vehicles or mine operators.
- Among others.

Considering the information and communication technologies (ICTs) linked to the Industry 4.0 era, for data collection and transmission, there are similarly several alternative devices that underground mine operators can use, from Ethernet networks to any number of wireless systems [16,17].

### 1.3. Why Underground Mine Wireless Monitoring Is More Cost-Effective and Mark a Road Map to Sustainability

**Figure 2:**
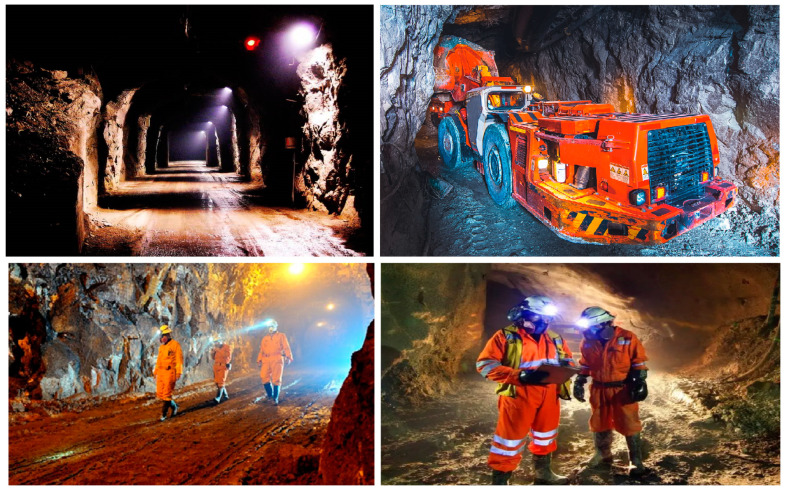
Typical environmental conditions in a medium- or large industrial-scale underground mine.

Underground mines are considered one of the riskiest facilities in the human industry, and for this reason, the mining local regulatory authorities are demanding mining companies apply the best available technologies (BATs), the best available practices (BAPs), and the best environmental practices (BEPs) [18,19] (see Figure 2). Today, monitoring systems connected to sensors with the use of cables are still used in underground mines. However, these systems have disadvantages such as the following: (i) wear of the cables due to time of use in the conditions of an underground mine, (ii) as the underground mine expands, the cable system must be expanded as well, increasing costs, and (iii) in the case of accidents (dust or gas explosions), the cables break, and the system does not function properly [13,14,20]. As a result of this, the emergence of wireless monitoring and communication systems is crucial to solving these difficulties of wired monitoring systems.

When instrumentation was first introduced to the mining industry, it relied on wired systems. Today, wireless monitoring can replicate the physical design criteria of these wired systems by using links to transmit data. Wireless systems are becoming increasingly common in various applications due to their substantial operational and cost advantages over wired systems. Some additional reasons of why wireless monitoring is more cost-effective include the following:

- No wiring required: a wired configuration may require a large distance equivalent to meters of cable to connect different sensors or gauges. With installation costs ranging from USD $15 to USD $30 per meter, a wired system can become very expensive if connections span thousands of meters. Wireless systems, powered by batteries, photovoltaic panels, or local power, eliminate the need for conduits and hardwiring, thereby removing these costs.
- Significant reduction in the installation costs: installing a wired system with 75 or 100 m of conduit can cost up to USD $2000, while a comparable wireless system might cost only a few hundred dollars per sensor or gauge, according to the vendor and application characteristics. Wired systems require new cables, trenching, hardware, and labor for repairs or reconfigurations. In contrast, wireless systems can be easily expanded without additional hardware. Furthermore, a wireless system can be configured in the office on the ground, reducing on-site labor by 60% to 80%.
- Effective operation in complex terrains: wired systems may face limitations in areas where cables cannot be run, such as open-pit mines, underground mines, or across properties not owned by the mining company. Wireless monitoring systems, using industrial transceiver nodes, provide powerful, long-range data transmission, maintaining signal strength through challenging terrain, structures, or adverse weather conditions. These systems can operate unattended for years, even in harsh environments, such as extreme hydrometeorological events caused by climate change.
- Complete collection of data without data loss: If a wired system fails due to issues like humidity conditions, corrosion, cut wires, or other adverse conditions, operators may not be alerted to the failure, leading to suboptimal operations or problems until the instrumentation is restored. Wireless systems, however, can be programmed with communication link alarms to alert operators if data transmission is interrupted. Preventative maintenance can be more effectively managed through wireless diagnostics, avoiding most problems.
- Versatility with different sensors: wireless monitoring configurations with open physical design criteria allow users to integrate various types of sensors to monitor different parameters. This flexibility means that sensors can be added or removed as needed to measure parameters, such as deformation, humidity, temperature, water level, and gasses, among others. Users can select the most appropriate sensor for each application, consolidating data from different sensors into a single interface.

Wireless control configurations are more cost-effective and versatile than traditional gauges and wired systems, addressing challenges that wired systems simply cannot overcome. In this sense, an underground mine activity that is more cost-effective and sustainable depends on the implementation of wireless sensor monitoring systems [21,22]. According to that, several factors, including the project lifetime, vehicle fuel and maintenance, number of sensors, and cabling and/or geotechnical engineering team costs, need to be analyzed. Additionally, is the number of mine operators deployed and the estimated daily rate of field geotechnical engineers, including insurance, and also, the number and cost of wireless monitoring equipment deployed. Finally, another issue to consider is the number of days allocated for monitoring and maintenance per month or year [23].

### 1.4. A New Era in the Technological Context of Mining

The conception of a smart mining business model must revolve around the integration of its entire value chain from start to finish and implies the migration to a productive model associated with Industry 4.0, in which technologies such as the IoT, advanced analytics, digital twins (DT), robotics, and the use of AI are key players in achieving better products and more efficient processes, in addition to generating sustainability in the territory [24]. This is how in the current Industry 4.0 era of the wireless monitoring system can be implemented in all the critical points of an underground mine that are necessary, where an efficient data transmission system can be implemented with the use of repeaters and gateways or boosters located at regular intervals in tunnels and galleries of the underground mine [25,26]. With the emergence of the IoT, which can be understood as the ability to extend network connectivity to objects other than computers, such as equipment, sensors, and everyday items, these devices are allowed to generate, exchange, and consume data with minimal human intervention, which in turn greatly expands the possibilities of monitoring operational and human risks in an underground mine environment [27]. Technological advances associated with the IoT expand the possibilities for capturing and transmitting real-time information related to the safety of people, variables in their environment, and the conditions of equipment in operation. In recent years, the development of so-called “wearables” (clothing, equipment, and other accessories that incorporate computers and advanced electronic equipment) has become widespread, facilitating the monitoring of biometric conditions in operators or environmental conditions in the workplace [28]. Some examples of the different categories of “wearables” include the following:

- Personnel tracking: use of proximity sensors (e.g., beacons) to locate front-line workers in the operation.
- Augmented reality and virtual reality: displays essential safety and maintenance data on glasses (e.g., equipment data, alerts, and procedure checklists, among others).
- Environmental monitoring: sensors and detectors to track key metrics (e.g., oxygen levels, heat, and hazardous gases, among others) and high-definition cameras to record procedures and provide real-time instructions when needed (e.g., when performing maintenance tasks underground).
- Biometric monitoring: devices containing sensors worn by the workforce to capture information about employee health status (e.g., heart rate, concentration, fatigue levels, repetitive biometric movements, and load weights, among others)

The different processes existing in an underground mine, from the exploration stage to the exploitation stage, provide a large amount of data and information, which, with adequate analysis, allows correct and timely decisions to be made. In this sense, the insertion of sensors into the underground mining environment plays a leading role together with the IoT, to capture data on the operation and maintenance parameters of its assets in a reliable manner, which makes it possible to measure their performance and thus determine points for improvement [29].

### 1.5. Scope of the Review

This article studies the feasibility of implementing a wireless sensor system for monitoring underground mines, considering the technology called LoRaWAN. The state of knowledge and practical implementation of this technology in underground mines is highlighted, considering a review of scientific publications available in Scopus and Web of Science.

The following research questions (RQs) are defined and studied in this review:

- RQ1: What are the main IoT technologies that allow the transmission of data collected by sensors implemented in underground mines according to Scopus and Web of Science publications?
- RQ2: What are the main publications in Scopus and Web of Science on the use of wireless sensor systems through LoRaWAN technology in underground mines?
- RQ3: What are the main characteristics, advantages, and disadvantages of a sensor system using cables and wireless for implementation in underground mines?
- RQ4: What are the main characteristics, potentialities, and limitations of each of the IoT technologies identified for implementation in underground mines?
- RQ5: What are the main design criteria to define the architecture of the LoRaWAN IoT technology to be applied in underground mines?
- RQ6: What should be the typical network topology of the LoRaWAN IoT technology to be applied in underground mines?
- RQ7: What are the sensors to be used with the LoRaWAN IoT technology to be applied in underground mines?
- RQ8: What are the technological advances, knowledge gaps, and future perspectives of LoRaWAN IoT technology to be applied in underground mines?

Finally, the following content structure was defined to communicate the main ideas of this review: Chapter 1: Introduction, Chapter 2: Literature review, Chapter 3: Comparison of Different Technologies for Wireless Monitoring in Underground Mine Environments, Chapter 4: Underground Mine LoRa Wireless Monitoring System, Chapter 5: Discussion of Results, and Chapter 6: Conclusions.

## 2. Literature Review—IoT Sensors and Wireless Connectivity System Applications

Next in the following chapter, the literature review is presented, where first the implemented research methodology is made known and then the current state of the art of the investigated topic is exposed.

### 2.1. Research Methodology

**Figure 3:**
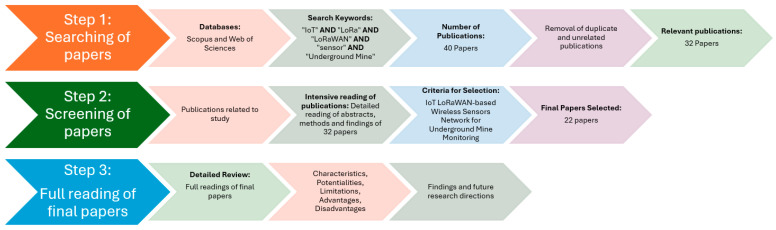
Literature review methodology applied.

To accurately study the literature on this research topic, this paper adopts a 3-stage methodology to identify and select publications closely related to the topic. The study process involved an initial literature search in the Scopus and Web of Science databases, followed by screening and a final content analysis. The methodological process for carrying out the literature review for this research is illustrated in Figure 3.

#### 2.1.1. Preliminary Search

Considering the first stage of the method used in this research, the period of the bibliographic search is from 2017 to 2024. The databases used for the bibliographic search process were Scopus and Web of Science (WoS), because they are some of the most complete, popular, and recognized databases in the world today. The use of other specialized databases is beyond the scope of this research. Another important criterion to mention is that these databases store quality articles, which implies a detailed description of the case studies presented, pointing out their advantages and disadvantages. An exhaustive search was conducted in the “Title-Abstract” field in Scopus and the “Subject-Abstract” field in WoS, respectively.

#### 2.1.2. Literature Screening

To ensure the quality of the reviewed publications, the literature screening phase was divided into 3 steps: (i) an initial search considering the keywords of this research, yielding this first search of some 40 publications, (ii) then, the types of publications were restricted to “review articles” and “articles”, and duplicates were eliminated, resulting in a preliminary selection of 32 documents, and (iii) the literature initially screened was subjected to a rigorous and detailed secondary screening. A careful examination of the abstracts, results, and conclusions identified 22 publications as relevant for this review. A summary of searching index considered in the literature search method according to the mentioned databases is detailed in Table 1. The number of publications found is low because this research topic corresponds to a relatively new technology in the mining industry. Consequently, a total of 22 publications were finally included in the review of this study.

#### 2.1.3. Literature Study

At this stage, the ideas are synthesized, and then, the selected publications are thoroughly analyzed. First, existing techniques and experiences are summarized. Second, the basic architecture structure of the IoT LoRaWAN wireless sensor system for underground mines is proposed, and the most feasible topological networks to use are analyzed. Finally, the types of sensors currently commonly used in underground mines are reviewed, the current state of development is analyzed, knowledge gaps are identified, and possible future research directions are proposed.

### 2.2. State-of-the-Art

A review of the scientific literature in Scopus and Web of Science databases has been carried out considering the implementation of wireless sensor networks in underground mines. As a result of this review, 22 publications have been found that are related to this research topic. Table 2 presents some aspects presented in these publications such as the following: (i) title, (ii) authors, (iii) sensors used, (iv) wireless connectivity system considered, (v) monitored parameters, (vi) implementation status around use in the mine and (vii) cost information.

Considering the publications reviewed in Table 2, it is possible to define a wireless sensor network (WSN) as a system that uses sensor nodes capable of processing data that work collaboratively. WSNs play a vital role in the IoT, increasing the awareness of specific environments and linking physical and virtual objects, using WSNs to connect the sensor nodes. In the IoT context, sensors and devices collect real-time data regarding production processes, equipment, the supply chain, and energy consumption. These data are analyzed to make decisions, predict maintenance, and optimize production. For example, advanced air quality monitoring techniques, especially in network design and space-time resolution, are applied in cities and in underground mines. It can offer additional insights into the experience acquired, challenges, and solutions related to monitoring networks, complementing the focus on IoT and machine learning [47].

This literature review shows that when the underground monitoring system is connected to the IoT, IoT objects exchange data to offer useful services. Short-range wireless technologies, such as Bluetooth, Wi-Fi, and ZigBee, were implemented in small-scale networks in the early stages of IoT. Although some mining operations still use Wi-Fi and Zigbee for data transmission, there are some limitations in terms of the signal range, but their costs are significantly lower in the long term than wired solutions. This is mainly due to the lower maintenance cost during its useful life [32,37]. Given the range limitations in Wi-Fi and ZigBee data transmission, there are other alternatives with a longer range. They are LPWAN (Low Power Wideband Network) technologies [23] such as the following:

- Narrowband Internet of Things (NB-IoT),
- Sigfox,
- Category M (CAT-M),
- Long Range (LoRa),
- Long-Term Evolution (LTE).

These technologies can be used on a large-scale IoT to effectively and reliably transmit signals over wide areas. Therefore, wireless monitoring in complex environments, such as underground mining, is now viable at a low cost [14].

According to Table 2, field monitoring systems in underground mine environments, most studies on mines were classified as atmospheric or geotechnical monitoring. In wearable systems, the health status of the mine operators was an important consideration, in addition to the environmental conditions of the mine. Also, it is possible to identify that the largest number of publications is made in Korea with 8 and the countries of the northern hemisphere are the leaders, also highlighting Germany, India, China, and Japan. On the other hand, in the southern hemisphere, Australia stands out with the highest number of publications with 2, followed by Brazil, Chile, Colombia, Ecuador, and Zambia. 

Additionally, observing Table 2, it is possible to mention that several underground mines have begun to implement connectivity through LoRaWAN. LoRa is a wireless technology that uses spread spectrum modulation. The use of this type of modulation allows for better noise tolerance and allows for long distances to be reached with very low energy consumption [23]. LoRa is the protocol at the physical layer level. LoRaWAN is the network-level communication protocol that runs on top of the LoRa physical layer. The LoRaWAN communication protocol is open, allowing many manufacturers to develop devices and thus reduce their costs [23]. In a similar way to an Ethernet network, the authors could say that LoRa is the cables that connect the devices in an Ethernet network and LoRaWAN is the communication of the devices at the level of the MAC address and the network IP address of the devices in the Ethernet network. LoRaWAN, for example, has major limitations in data transmission speed and does not have a standardized transport protocol, meaning it has some time limitations between consecutive transmissions. This issue needs to be studied more closely by developing demonstrative tests, to enhance the advantages of LoRaWAN further. In this article, the authors will refer to IoT LoRaWAN-based WSN as the set made up of LoRa and LoRaWAN (physical layer and communication protocol).

For a correct implementation, it is crucial to understand the propagation properties of data with IoT LoRaWAN-based WSNs within the underground mine. In this sense, it is necessary to study the propagation of data within the underground mine with measurements, both in a straight line without obstacles and in areas with obstacles (curves, corners, or changes in direction). It is fair to mention that information or publications on data propagation with the use of IoT LoRaWAN-based WSNs in underground mines are quite scarce and rare to find in scientific journals (Kumar et al., 2023 [21]; Branch, 2022 [22]; Branch and Zhao, 2020 [30], Duarte et al., 2022 [30,37], and Musonda et al., 2024 [23]).

This shows that the IoT LoRaWAN-based WSNs applied to underground mining is a topic that is still poorly studied worldwide and requires greater dedication from university researchers and support from the mining industry for its implementation in the field. In this sense, in a high percentage of the cases studied, the costs of IoT systems are not specified; only one publication (Pedrosa Santos et al., 2022) [39] mentions the costs.

## 3. Comparison of Different Technologies for Wireless Monitoring in Underground Mine Environments

The next section of this review presents an overview of wired and wireless sensor systems and describes a comparative analysis of cutting-edge wireless technologies.

### 3.1. Understanding Wire and Wireless Monitoring System Used in Underground Mines

**Figure 4:**
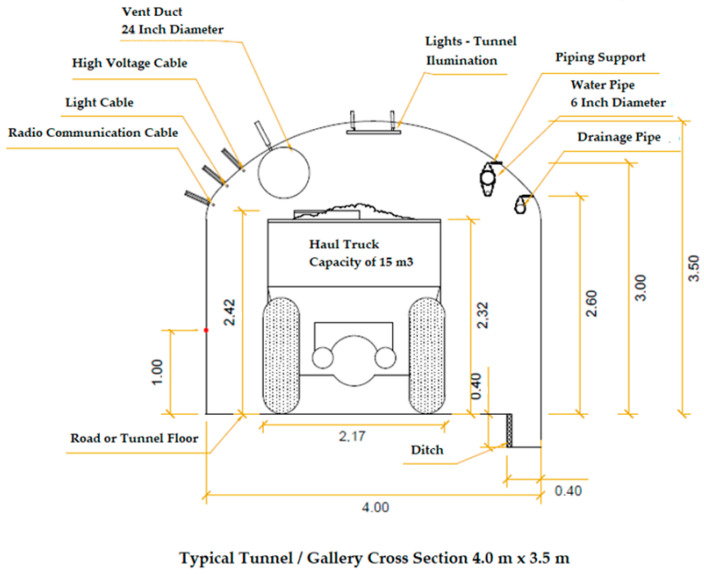
Typical tunnel/gallery cross-section in a conventional underground mine with the use of cables. All units are in meters. Adapted from [20].

To understand wireless data transmission on the way to a digital underground mine, it is necessary to understand how data transmission is carried out using cables, which is currently the most popular option in underground mines. Also, it is possible to measure the advantages and disadvantages of each of these alternatives. In this sense, to transmit the data captured by a monitoring system and make it available in real-time, some communication system technologies with wire and wireless are more popular:

- Fiber optic and/or coaxial cable (conventional wire system): communication systems based on fiber optics and/or coaxial cable have been used routinely in the mining industry. These systems’ advantages are (i) they allow the automatic control of variables, (ii) they allow real-time diagnosis, (iii) they offer stable operation with low maintenance, (iv) they allow control during post-operation, (v) they can transmit data over long distances, and (vi) they can transmit large amounts of data (high transfer rate compared to other wired systems) [13,14,20] (See Figure 4).
- Radio communication (non-conventional wireless system): these systems allow communication at anytime, anywhere, and on any device. Their advantages include (i) simultaneous communication to several receivers and (ii) the capacity to transmit early community warnings [42].

The wireless technology of the data-acquisition system eliminates the need for expensive cabling and manual monitoring in underground mine environments. Laying cables in a mine requires trenches and cable protection against issues, such as mine settlements. New sensors must be added as the underground mine grows, again requiring expensive cable installation. A wireless system provides data from sensors in near-real time, versus manually collected readings with a more sporadic periodicity and vulnerability to human error [23].

### 3.2. Comparative Analysis of IoT Technologies Applications in Underground Mining

Recent wireless monitoring platforms use nodes to collect readings from each sensor on an underground mine. The wireless nodes then send the information to a central gateway. This gateway, in turn, relays the data to an industrial server via an Internet connection. From there, the data can be analyzed in near real-time (phone apps, computer dashboards, etc.) and passed on to third-party applications for management and compliance reporting [30].

Table 3 presents a summary considering a comparison of different technologies for wireless monitoring in underground mine environments:

According to Table 3, most wireless connectivity systems depend on local area networks (LANs) based on well-known technologies, such as Zigbee, Bluetooth, and Wi-Fi, among others. These are mostly adequate for communication across distances of approximately 100 m, and so they are not suited to underground mine environments. For the other hand, considering 3G, 4G, and 5G, which are cellular technologies, these can reach longer distances and allow for frequent data collection. A challenge for underground mine environments of this kind of technology is that they have high power consumption rates [48]. To reduce power consumption, it is common for data to be sent only once a day or once a week. For this reason, cellular technology is not suitable for real-time or near-real-time data acquisition in underground mines. Finally, there is a low-power WAN with communicating devices such as LoRaWAN, Sigfox, and LTE-M, among others, which stands for long-range wireless communication and addresses the range and power consumption issues seen in LAN and cellular technologies. With this low-power, wide-area network technology, data can be transmitted over large distances with reduced power. Batteries can last very long as devices only “wake up” when they must read and transmit data. Also, for example, LoRaWAN technology does not depend on a service provider infrastructure, allowing users to deploy private networks and have greater control of their assets [49].

As follows, Table 4 shows a comparison, in detail, of the main technologies for wireless monitoring in underground mine environments considering technical specifications.

According to Table 3, it is possible to observe that LoRaWAN technology has a significant competitive advantage against other alternatives considering underground mine environments. In this context, in the following sections of this review, a proposal for the recommended design criteria related with architecture, network topology, and sensors to be implemented in a IoT LoRaWAN-based WSNs will be presented.

## 4. Underground Mine IoT LoRaWAN-Based WSN Monitoring System

Below in the following paragraphs, the definition of the main design criteria of an IoT LoRaWAN-based WSN monitoring system for use in underground mines is presented, considering the following: (i) architecture, (ii) network topology, and (iii) typical sensors for applications in underground mine environments.

### 4.1. Definition of Design Criteria for IoT Sensors and Wireless Connectivity Ecosystem Network Architecture

**Figure 5:**
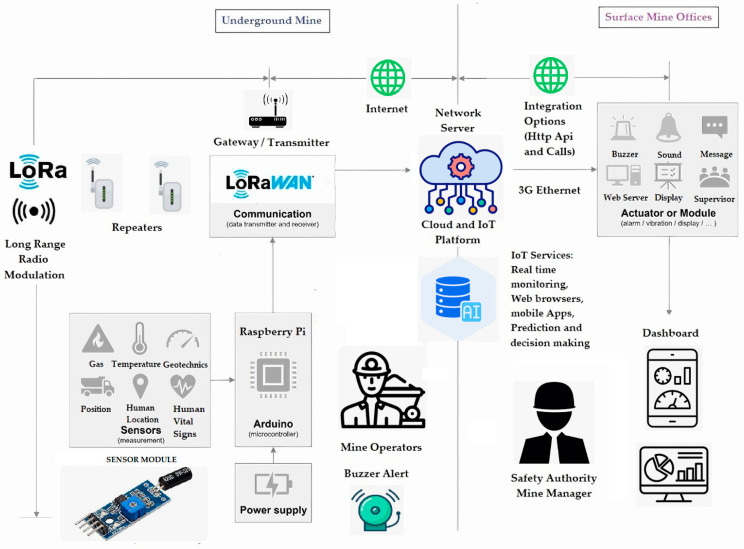
Schematical diagram of IoT and LoRaWAN architecture wireless safety monitoring system.

The design criteria of the IoT sensors and the wireless connectivity ecosystem system are key to defining the network architecture. In that sense, the linkage among sensors, wireless transmitters, data storage cloud, data processing method, and data visualization tools, among others, must be defined. Figure 5 shows a diagram of the recommended architecture to implement the LoRaWAN protocol in a typical underground mine project. In this figure, you can see the components of the system to be installed inside the underground mine and the components to be installed on the surface or outside of the underground mine:

The proposed architecture shown in Figure 5 includes wireless devices and software for monitoring key parameters in underground mine environments. Once sensors/nodes are connected to a wireless LoRa unit and the system’s gateways are properly installed on underground mine tunnels/galleries/ramps, they are ready to receive, store, and send data. Data can be available through a web interface, which enables users to monitor the underground mine infrastructure remotely [54].

The sensors usually used are of the low-cost type and can be adapted to Arduino or Raspberry Pi processing systems, popularly known and widely used in the industry today [14]. The mine operators can set sampling rates, check radio coverage, and view the real-time data captured by the sensors. Each sensor unit for monitoring gas, temperature, geotechnics (roof stability), vehicle position, human location, and human vital signs, among others, can be a smart node within the WSN. The wireless units are capable of reading at a sampling rate of once every 30 s to once every 24 h, depending on the requirements [55].

Wireless sensor networks (WSNs) considered in this architecture can be easily expanded by adding new units to the network. The IoT LoRaWAN-based WSN protocols have been designed to be highly scalable, with a single gateway managing up to 1000 nodes. Network latency and packet loss ratios are reduced due to the robust performance of protocols. An important aspect of the IoT LoRaWAN-based WSN system implemented in underground mines is the use of repeaters, which are necessary to avoid a reduction in signal transmission due to physical obstacles, such as walls, curves, or ramps [56].

The IoT LoRaWAN-based WSN device network is compliant with CE and Federal Communications Commission certifications and operates with ultra-low energy demands in industrial, scientific, and medical (ISM) radio bands. The IoT LoRaWAN-based WSN system’s low-power components remain in sleep mode and are only activated at predetermined times, ensuring a life span of up to 10 years [57].

Data access and compatibility with data visualization software is another important consideration for monitoring and understanding the key performance indicators (KPIs) of underground mine functioning. In a monitoring plan, several monitoring methods will be required: geodetic techniques, vibration monitoring, wireless solutions, and so on. Usually, these data are presented in information management and visualization software. It is therefore important to see how data from the wireless system may be integrated into the project software [58].

This system allows you to have alerts or alarms considering buzzers that notify mine operators within the underground mine, as well as send notifications by mobile phone sounds or messages to operators and managers who are on the surface or outside the underground mine. In this way, a real-time decision-making environment is promoted that allows for improving productivity, reducing accident risks, and improving the sustainability of the work environment in underground mines [59].

Additionally, some authors define key issues to consider in the network design criteria for the LoRaWAN system architecture, for example, the following:

- Kumar et al., 2023, [21], in the research entitled Development of LoRa Communication System for Effective Transmission of Data from Underground Coal Mines, mentions the following idea: “The LoRaWAN network’s topology is star-of-stars in terms of its own architecture and is comprised of three primary parts network servers, gateways (GWs), and end nodes. End nodes require gateways (GWs) to connect to the network server (or data server)”,
- Branch, 2022, [22], in the research entitled Measurements and Models of 915 MHz LoRa Radio Propagation in an Underground Gold Mine, mentions the following considerations: “Research into LoRa has mostly emphasized its applications. LoRa is particularly well suited to agriculture. Farms where distances are a maximum of a few kilometres match LoRa’s single hop star architecture well. Agriculture applications have included soil moisture monitoring, livestock location and behaviour, and some aquaculture applications”.
- Aziz, 2020, [38], in the research entitled A Study on Industrial IoT for the Mining Industry: Synthesized Architecture and Open Research Direction, affirms some key issues as following: “The layered architecture addresses the current challenges in the mining industry by offering a remotely controlled, automated, and interoperable environment to improve communication, data access, and data management. The architecture considers each mine site as an IIoT edge, which addresses all serious and complex issues by local edge gateway, name a few as asset management, monitoring and diagnosis, provisioning and deployment, and interoperability between different systems and devices. There is a need for an IIoT architecture for the mining industry that follows the industrial guidelines defined by standard bodies and adopted by a large number of vendors”.

Limitations and critical analysis of LoRa are explained in detail in the discussion chapter.

Finally, the development of a reference architecture for the integration of mining processes in an underground mine must propose an alternative for the interoperability of its components and the supervision of operations, with global visibility of the processes. The architecture must consist of at least three layers: (i) an integration layer, where the access mechanisms to the data sources and applications of the underground mine are established; (ii) a data model layer, where the physical assets of the mining business are described; and (iii) a management layer, in which, through multi-agent systems, its value chain processes are executed and supervised.

### 4.2. Definition of Network Topology for IoT Sensors and Wireless Connectivity Ecosystem

**Figure 6:**
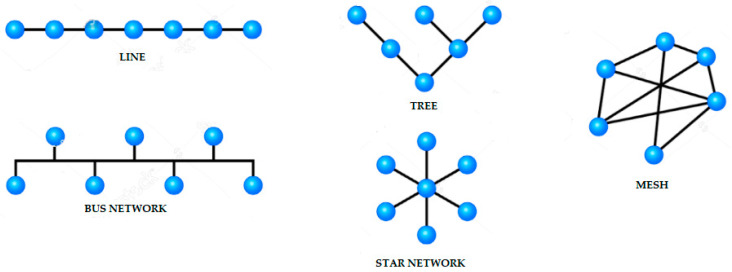
Typical network topology types for application in mining project environments.

**Figure 7:**
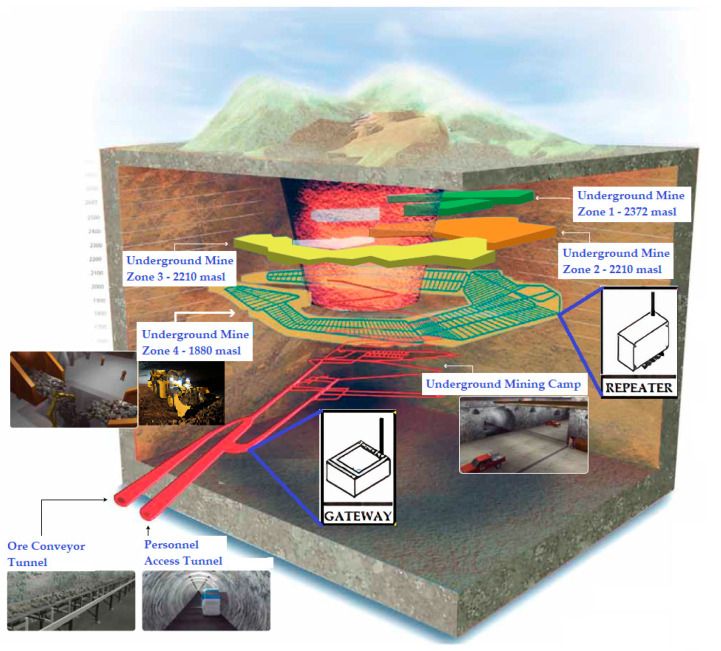
Panoramic view of a typical configuration of tunnels and galleries in an underground mine. Positioning of repeaters and gateways in an IoT LoRaWAN-based WSN system on an underground mine environment.

**Figure 8:**
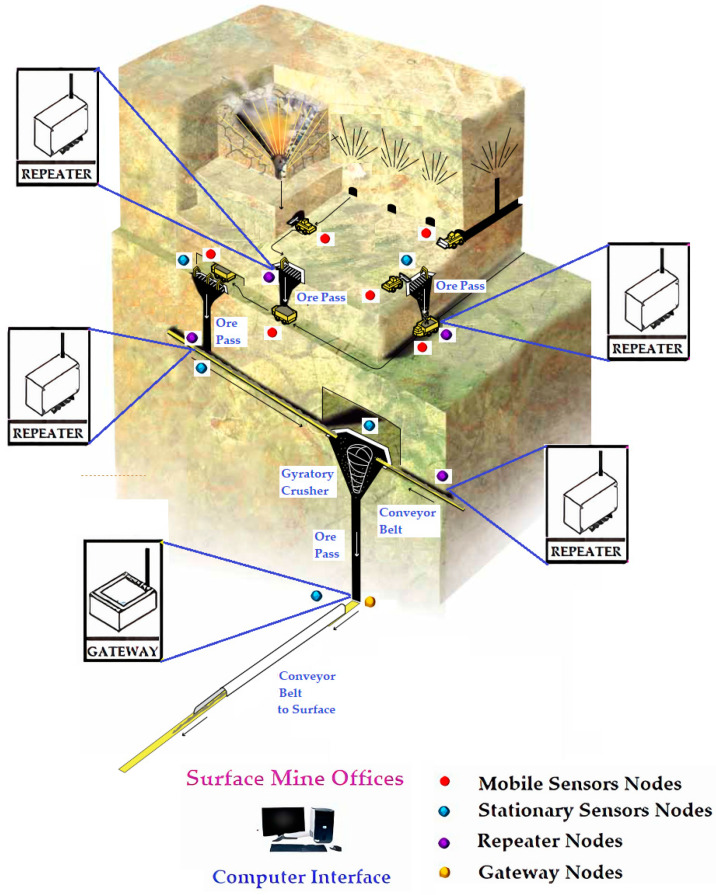
Layout view of the positioning of sensor nodes, repeater nodes, and gateway nodes considering IoT LoRaWAN-based WSN technology for safety monitoring in an underground mine.

Due to the complex conditions of wireless communication in underground mines, higher requests are being made for the stability and reliability of the wireless sensors network (WSN). Based on network connectivity and coverage, one can optimize the network topology and extend the lifetime of the whole network. Figure 6 and Table 5 show that by adjusting the transmitting power of nodes and selecting some nodes as a backbone, this can deal with data processing and transmission based on certain principles:

The topology configurations presented above in Figure 6 and Table 4 can be applied in mining projects. Surface mining and underground mining infrastructures used the following topologies:

- Surface mining projects: when connecting sensors and the Internet of Things in surface mining infrastructures, the IoT LoRaWAN-based WSN system can be implemented through a single loop with a Star-type topology. This means that when it is required to install a real-time monitoring system in remote areas that are difficult to access on the surface, this technology can do so without the use of repeaters. When there are no geographical obstacles or infrastructure barriers, IoT LoRaWAN-based WSNs in these cases can have a range of 15 km of network coverage, more than any other comparable system. This makes this technology ideal for monitoring large-scale civil infrastructure, such as mining.
- Underground mining projects: when connecting sensors and the Internet of Things in underground mining infrastructure environments, the IoT LoRaWAN-based WSN system can be implemented through several loops with a Tree-type topology. This means that when it is necessary to install a real-time monitoring system in areas with physical obstacles with complex topography with the presence of walls, curves, or long tunnels, this technology can do so in those intricate topologies with the use of repeaters. When there are topographic obstacles, ramps, walls, galleries, tunnels, curves, and ventilation shafts, among others, IoT LoRaWAN-based WSNs in these cases can have a range of 10 km of network coverage, more than any other comparable system. The key component of this system is the repeaters due to the linear nature of communications in these environments, which allow the signal from a node to be received and retransmitted (see Figure 7). This is how repeaters can be strategically installed in the network of tunnels and galleries of the underground mine to overcome all types of physical obstacles (see Figure 8). This makes this technology ideal for monitoring complex infrastructures in large-scale inhospitable environments, such as underground mining.

Figure 7 and Figure 8 show that is very important to positioning the repeaters strategically to promote the adequate wireless transmission of data to not have difficulties with physical barriers. A computational simulation is recommended to carry out and, in this manner, study the performance of the IoT LoRaWAN-based WSN and define the optimal configuration of sensors, repeaters, and gateway locations.

### 4.3. Definition of IoT Sensors to Be Implemented in Underground Mine Environment

The main criteria design for the definition of sensors to be applied in an underground environment are (i) safety and (ii) geotechnical stability. In this sense, each sensor unit for monitoring safety is defined considering the following: (i) the presence of harmful gas, (ii) environment temperature, (iii) security control in explosive stores, (iv) human location—tracking of people at all important control points of the mine, (v) guarantees air flow, (vi) monitoring of fans and controlling toxic gases—Machine to Machine (M2M), (vii) vehicle collision avoidance and personnel safety, (viii) control the use of personal protection elements (PPEs), (ix) control of water and energy resources, and (x) human vital signs, among others.

On the other hand, each sensor unit for monitoring geotechnical stability is defined according to (i) roof stability, (ii) supervision of tunnel conversion, (iii) control of anchorage bolts, (iv) visibility of the operation on the mine work front, (v) vehicle position—vehicular tracking of haul trucks, scoops, drills, and personnel trucks, (vi) control of traffic lights inside the mine, (vii) micro-seismic control systems, (viii) mobile control in underground operation, (ix) control of groundwater levels and automatic control of water pumps, and (x) centralized and distributed control of the operation, among others.

Considering the geotechnical stability aspects, with sensors, it is possible to closely monitor the tension and stress of the walls of the advancing face in the production phase by continuously monitoring the vertical movements and convergence of the tunnel, such as the following [48]:

- Ground movements: extensometer-type sensors can monitor ground movements inside the tunnels using wireless digital data recorders to transmit the data.
- Convergence control in underground mine infrastructure: wireless laser-tilt-type sensors can support the supervision of convergence in access ramps, tunnels, and galleries.
- Monitoring the stability of the pillars between chambers: sensors allow for control of the load cells that can be installed on the rock pillars in order to evaluate the accumulation pressure due to surrounding excavations.

Furthermore, regarding the aspects of geotechnical stability, using sensors it is also feasible to keep the structural health of the tunnel support systems stable, by monitoring the load and movement of the soil and rock, such as [58].

- Monitoring of the load on the anchor bolts in the rock: through sensors placed in the rock corresponding to load cells connected to a variety of wireless recorders, which can be located in places where the structures are at risk of rock fall.
- Study of soil movements in excavation areas: it is possible to safely connect several bolts using wireless digital data loggers and thus transmit data from the sensors.
- Control of cracks in tunnels: through the use of cracking meter-type sensors, it is possible to monitor the evolution of surface cracks in tunnels and thus collect the data using digital recorders.

The underground mine can also require the reading of data from chains of digital in-place inclinometers using a battery-powered node. In addition, two-in-one sensors and nodes, such as wireless tiltmeters or laser distance nodes, may be considered. Sensors, repeaters, and gateways need to have water tightness, robustness in extreme environments, and resistance to extreme temperatures. The nodes should ideally not require re-casing. For example, IP67 waterproof units can be used, which are rated and tested from −40 °C to +80 °C.

## 5. Discussion

### 5.1. Technological Advances

Considering the current time to the year 2024, it is possible to say that IoT technologies, long-range wireless transmission, and smart sensors are already about to be implemented. New case studies are appearing day by day, where little by little, more experience is being acquired and the evolutionary development of these Industry 4.0 technologies is being expanded, linking them to mining. This means a great opportunity for the mining industry, where the technologies already exist, can be learned, and implemented. In this sense, different advances exist where wireless monitoring systems have improved underground mine environments’ safety.

First, IoT LoRaWAN-based WSNs enhance the understanding of the most critical potential failure mechanisms: accidents or geotechnical failures in underground mines can stem from various sources, such as fires caused by machinery, weakening of tunnel supports, abnormal gas concentrations, or drainage system failures that increase groundwater levels, among other factors. Inadequate monitoring systems can further contribute to these risks. Traditional manual or wired in situ monitoring does not provide miners with sufficient data to effectively control the key factors that contribute to accidents or geotechnical failures. This lack of data greatly increases the risk in an underground mine. In contrast, the large volume of data and the frequency with which they are collected through low-cost wireless sensor monitoring solutions form a robust foundation for effective risk prevention and creating digital twin models that help understand critical geotechnical failure mechanisms.

Second, IoT LoRaWAN-based WSN identifies situations that could potentially trigger accidents in underground mines: real-time data collection ensures that miners have continuous information about the status of all underground mine conditions. Wireless monitoring systems enable the implementation of corrective measures at the earliest possible stage, ideally before any incident occurs. This approach is crucial not only for reducing risks and saving lives but also for protecting a business’s profit margins. The costs associated with post-incident remedial action and mine downtime are significantly higher than those for predictive maintenance activities.

Third, IoT LoRaWAN-based WSNs serve as a tool for planning the growth of the underground mine: the accumulation of data through wireless monitoring devices is vital for future planning and expansion of the mine. With wireless monitoring solutions, miners can use reliable data to develop models for more accurate planning regarding potential future changes and incidents in the context of the mine’s growth.

Fourth, IoT LoRaWAN-based WSNs provide a solid basis for establishing and implementing appropriate responses to failures involving human hazards: equipped with the capability to model future incidents and respond predictively, miners can develop standardized responses for recurring incidents and establish contingency plans for emergencies.

Fifth, IoT LoRaWAN-based WSNs facilitate long-term monitoring with minimal maintenance: a monitoring system that is both durable and requires minimal maintenance is critical in underground mine environments. This involves ensuring that the equipment can withstand harsh conditions, has a long battery life to avoid frequent replacements, and is capable of long-distance transmission to consistently relay data from hard-to-reach areas over extended periods.

Finally, some merits to consider of IoT LoRaWAN-based WSN technology for use in underground mines are the following:

- Low power: the devices are optimized to operate in low-power mode and can last up to 10 years with minimal maintenance.
- High capacity: IoT LoRaWAN-based WSN servers can handle millions of messages from hundreds of nodes, resulting in significant operational cost savings.
- Comprehensive security: IoT LoRaWAN-based WSNs guarantee secure communication between the end device and the application server. It is a reliable system that can transmit data from sensors at the underground level of the mine to the surface.
- Early detection: the system can detect an anomaly and give a warning signal to the surface.
- Operational robustness: the solution can withstand adverse weather conditions and is ideal for underground environments and high altitudes.
- Adaptability of on-premises or cloud server configuration: you can choose between a single-network on-premises server configuration or a multi-network cloud configuration with expanded IoT LoRaWAN-based WSN features depending on the characteristics and needs of the underground mine.
- Flexibility of network topologies: networks used through IoT LoRaWAN-based WSNs can be a single-hop configuration or a multi-hop configuration as required by underground mine conditions.
- Cost-effective solution: IoT LoRaWAN-based WSN is a low-cost, robust solution with low power demand and long range.
- Mobility: as it is a wireless communications system, it is possible to easily adjust and move the sensors.
- Reliability: as a wireless communications system is used, it can monitor any corner and corner in an underground mine compared to a wired system.

### 5.2. Knowledge Gaps

There is a significant gap in the number of scientific publications regarding the application of sensors in wireless networks using IoT LoRAWAN. According to the search results of this research, only 22 publications have been published to date that discuss this research topic promoting the digitalization, productivity, and sustainability of underground mining activity.

There is an important gap with this Industry 4.0 technology in mining, which is related to the training and learning of people. Not all people understand and are willing to implement these new technologies. There are several reasons, and among them, we can mention the following: (i) fear of the new along with opposition to change, (ii) misinformation, and (iii) lack of formal training courses, among others. Correct wireless monitoring requires a deep understanding of the underground mine functioning, as well as the principles of the measurement and instrumentation deployment. This requirement for deep specialist knowledge is considered a gap because not all underground mines have the chance to always have this professional on-site.

Sensor monitoring is usually not connected to centralized information technology (IT) and operational technology (OT) systems in underground mines. Instead, data collection and processing are normally performed locally at each mine. This can hamper the mine operator’s ability to carry out complex analytics in real-time and works against the implementation of standardized designs.

The widespread use of smart sensors in underground mining is lacking, whereby the definition of this type of sensor is a set in which one or more sensor elements and some signal conditioning instruments are arranged in the same physical unit, that is, the combination of an analog or digital sensor with a processor, a memory, and a network controller on the same board. A smart sensor provides information to the data obtained to support decision-making and distributed processing. These sensors allow the equipment to self-regulate its consumption because it makes it work at its optimal capacity.

For the other hand, the lack of standardization and reference architectures across underground mines does not favor scale economies or integration capabilities, so in some cases, wireless sensor monitoring technologies have tended to be viewed as costly and hard to work with.

In this sense, interoperability is a concept closely linked to digitalization and is essential for progress toward the mining of the future. Without digitalization, in the sense of changing information from analog to digital format, data exchange would be cumbersome. On the other hand, digitalization from the perspective of changing the current operating model to one based on the use of digital technologies would also be impossible to achieve. Thus, without interoperability, mining equipment, and computer systems would remain stuck in current data structures, which have hindered mining process improvement initiatives for years. In other words, interoperability is an essential requirement to maximize the capabilities and benefits of mining automation, digitalization, operational integration, and data analysis initiatives; and it can also be a huge driver of innovation since mining 4.0 will require an extraordinary level of collaborative teamwork between people, software, and machines.

Another aspect to consider a gap is related to securing underground mine sites is a challenging task due to the complexity of the infrastructure, the variety of physical and digital components, the distribution of assets and types of machinery, and the large number of workers and stakeholders involved, among others. In recent years, mine operators have been adopting innovative information and communications technologies linked to the Industry 4.0 paradigm, but in some cases, they still use archaic or manual processes, thus making the protection of critical assets a costly and complex problem.

For other hand, security is central to the protocol implementation of IoT sensors and LoRaWAN wireless connectivity systems. Advanced encryption mechanisms must be studied and used to ensure that the solution is ideal for mission-critical applications in underground mine environments.

It is important comments in general terms that the LoRaWAN characteristic describes thee classes of functions: (i) Class A, low-energy bi-directional end devices; (ii) Class B, scheduled downlink transmission; and (iii) Class C, designed for always-on bi-directional actuators.

Class-A devices offer the highest energy efficiency, suitable for applications with infrequent communication needs, with brief receive windows after each transmission. Class-B devices provide predictable downlink communication latency by synchronizing with network beacons and enabling scheduled receive slots, maintaining moderate energy consumption. Class-C devices prioritize down-link latency over energy efficiency, offering continuous receiving windows for near real-time communication. End devices use a random-access transmission method (ALOHA), which allows them communication without the need for pairing with a specific gateway [61,62,63].

Class A is the class of LoRaWAN devices with the lowest energy consumption. Class-B devices are designed for applications with additional downlink traffic needs. These devices are synchronized using periodic beacons sent by the gateway to allow the schedule of additional receiving windows for downlink traffic without prior successful uplink transmissions [61,62,63]. 

The three classes can coexist in the same network, and devices can switch from one class to another. However, there is not a specific message defined by LoRaWAN to inform the gateway about the class of a device, and this is up to the application. In this sense, the latency level depends on the class considered in LORAWAN [61,62,63].

The authors recommend to study latency in LORAWAN systems carrying out technical feasibility studies considering the following parameters, downlink traffic and energy consumption, arise and the site-specific conditions of the mining project.

### 5.3. Future Perspectives

The mining industry is waking up to the fact that such losses will no longer be tolerated by investors, local communities, and other stakeholders. Until recently, dealing with this challenge was not easy. The only way to prevent incidents is by adopting a multidisciplinary approach to risk management, tracking diverse environmental factors, and forging links between geotechnical engineering and IT spheres of expertise, among others, all as cost-effectively as possible.

Taking changing field conditions into account is crucial because safety factors can change over time. Thus, it helps to have regular monitoring, with readings taken on a daily or even hourly basis. Until recently, addressing all of these points was a tall order. Now, though, the technology exists to connect virtually any sensor to any data visualization system, offering end-to-end linkage from information capture to alert communication in near real time.

Once installed, such equipment can provide reliable, low-cost monitoring for years on end, giving operators an unrivalled opportunity to spot potential failures before they happen. The technology could ultimately serve as the platform for a whole ecosystem of smart, sensor-based mining services and applications, from asset tracking to plant condition monitoring.

The following aspects need to be developed in the next years to improve the service quality of IoT LoRaWAN-based WSNs in underground mine environments:

- Providing a standardized open technology stack that is fully scalable and compatible with established corporate standards.
- Launching a range of robust, self-contained, Lithium-ion Battery-powered (LiB) wireless nodes that can read most existing market devices.
- Developing products that are compatible with visualization software and third-party devices.
- Interacting with regulatory frameworks and contributing to industry best practices.
- Designing tools to manage networks and data.
- Interoperability and integration with different ICTs.
- Scalability: starting with a demonstration system and moving to an industrial-scale system.
- Security: improving the safety of the systems against collapse or adverse climate conditions.
- Digital twins: more connections with simulation devices like digital twins are needed to provide different scenarios of underground mine operations.

The set of sensors and IoT must be linked to DT, where a digital twin is defined as a digital replica of real physical assets, processes, people, places, systems, and devices, which can be used for various purposes in an underground mine. Among its potential uses in the underground mine, it should be considered, for example, the inclusion of a learning digital twin (DT) for personnel from various engineering and geology disciplines, which will allow a rapid simulation of geological exploration options, planning, drilling, blasting, and process control.

The development of a global DT where all engineering/geology disciplines (knowledge/data), all stages (time), and all mining processes (activities) are incorporated will give mining companies with underground mines the ability to create digitalized versions of their components, which can be updated in real-time employing sensors or tags located in the physical equipment. The ability to create a digital representation of a physical asset in an underground mine can provide important data on the status of each asset. The data gathered can streamline an underground mine’s mining plan by predicting potential disruptions before they occur, thereby reducing, for example, the risk of an unscheduled stoppage in any of its processes.

According to the context of mining 4.0, IT/OT systems are no longer systems that flow through separate networks. Technologies, such as the remote operation of mining equipment, the IoT, and the Cloud, have made IT and OT networks converge to allow the monitoring, management, and modeling of mining production, which implies new challenges in terms of cybersecurity. From the perspective described, it is vital to proactively monitor data traffic within the networks in the operation, to detect threats that are in the process networks. There are various ways to detect these threats, which range from protection and analysis at the “endpoint” through detection and response systems, Log analyzers with malware detection intelligence, and the use of next-generation firewalls, which include the technology to generate the recognition of industrial cybersecurity protocols and allow for the detection of advanced or denial of service threats.

Three characteristics can be used to describe a network in radio communications technology: (i) range, (ii) data transfer rate, and (iii) power consumption. It is difficult to give equal importance to all three criteria because the laws of physics have clear limits on this: for example, LoRaWAN can transmit data over long distances with relatively little power, but at very low data rates. In that sense, some limitations affect the range of LoRaWAN that need to be considered, they are the following:

- Free space attenuation factor: doubling the distance, LoRaWAN’s free space attenuation increases by 6 dB. In addition to the power loss caused by the LoRaWAN range, the reflection, and refraction of radio waves off objects can also cause radio waves to overlap.
- Structural damping factor: the attenuation of radio signals as they pass through different obstacles affects the reception of transmitted signals and ensures that the signal range is greatly reduced.
- Fresnel zone factor: it is essential to establish as straight a line of sight as possible between the transmitter and receiver if an underground mine project wants to cover long distances effectively and get a good balance of power transmission. Certain areas of the space between the lines of sight of the radio transmission are Fresnel regions. Wave propagation will be negatively affected if there are objects in these areas, despite the usual visual contact between the transmitting and receiving antennas. For each object in the Fresnel belt, the signal level drops, and the LoRaWAN range is reduced.
- One of the main limitations of LoRaWAN is the relatively high latency. Networks and devices communicate with each other using data packets. However, these data packets are not always transferred immediately, as they consume battery power and network coverage. Latency is the delay time in data transfer after a transfer request is made. A low-latency device “connects” to the network more often than a high-latency device. For example, a smart sensor detects excessive deformation of support in an underground mine tunnel and needs to send an alert to the network. If this sensor has high latency, it does not transfer data to the network frequently, and it may take a few minutes before the network receives the alert. If the sensor has low latency, the network will receive the alert much sooner.

Finally, the main limitation of this research is the lack of sufficient real experience in implementing IoT LoRaWAN-based WSN technology in underground mines, mainly because it is a new technological tool, with little use in the mining industry and the development/maturation stage. In this sense, it is recommended to implement pilot tests in underground mines, to study the behavior and performance of the technology, and in this way, subsequently carry out a massive scale-up and use throughout the tunnel network of an underground mine.

## 6. Conclusions

Wireless monitoring systems provide a real-time, continuous flow of data enabled through reliable, long-distance, low-energy, and low-maintenance technologies. Using IoT sensors and LoRaWAN wireless monitoring systems is a key tool to properly monitor underground mine activities to prevent accidents and sustainable safety management.

Furthermore, mine assets are deployed across large and harsh environments, where they are vulnerable to a long list of human errors and natural disasters, increasingly including climate change hazards. For example, cellphone APPs enable mine operator users to set up and visualize the performance of each device, task, or machinery operation on an underground mine during installation, minimizing the need for highly trained technicians.

LoRaWAN (communication protocol) is based on LoRa (physical layer), where an IoT LoRaWAN-based WSN is the set of the physical layer with the communication protocol. It has been developed thinking about large-scale IoT communications where it is necessary: (i) due to their long-range capabilities, wireless devices are perfect for harsh environments or difficult-to-access sites like underground mine environments; (ii) transmit signals through physical barriers; (iii) minimize maintenance operations and site visits; and (iv) allowing massive scale with low implementation and maintenance costs.

One of the major comparative advantages of a LoRaWAN wireless connectivity system is the following: (i) a private and secure network since it does not require the use of public connectivity infrastructure to connect to the servers; (ii) versatility and flexibility of the servers, which can consider cloud usage options and the transmission of data from the gateways can be via a cellular internet connection, Wi-Fi, or Ethernet; (iii) it is feasible to configure an independent network, managed locally in the mining operation. This is how in an underground mine, different types of sensors linked to IoT LoRaWAN-based WSNs can be implemented to monitor various parameters. Then, IoT LoRaWAN-based WSNs will transmit the data collected from the sensors to gateways. These gateways have the flexibility of being connected via Wi-Fi or the wired Ethernet to the local area network of the underground mine administration, where the following servers can be located: network, union, and applications.

Data transmission is the most important factor in an underground mine environment, considering the selection of a wireless system because the distance between monitoring points can be significant. The monitoring WSN systems should be easy to deploy anywhere and not dependent on signal coverage. Usually, the costliest item in a monitoring infrastructure is cable protection. Through an IoT LoRaWAN long-range wireless system, an underground mine environment may save kilometers of cables and decrease installation and maintenance costs.

Another important consideration when evaluating a potential monitoring solution is the ability of the network to automatically adapt to changes in the sensor network setup. The WSN needs to be capable of updating itself and should not require manual reprogramming, and the network protocols must be designed to be highly scalable. Additional sensors must be easy to install and remove. 

From the academy, we also understand that this process of transformation towards a digital underground mine, being immersed in an environment of constant change and high uncertainty, is complex, both from the cultural and technological perspective. This uncertainty, however, can be managed through collaborative processes that allow the industry to address common problems and to signal the innovation ecosystem the way forward. In this way, the interoperability of the systems and the availability of the solutions and talent required promptly are ensured.

By having interoperability between different digital platforms in an underground mine, for example, operational management, safety management, and environmental management, it becomes possible to cross-reference and analyze data to predict possible risks, both physical and behavioral, and take the necessary actions to reduce and eliminate them.

Critical information infrastructure, such as an IoT LoRaWAN long-range wireless system, which includes facilities, networks, services, and physical and information technology equipment, for which the impact has a significant impact on the security and effective operation of the mining business, must be especially protected. For this reason, mining companies must design a reference architecture capable of dealing with events that may disrupt the normal development of their operations, so that they can adapt to natural phenomena, human interventions, or computer interferences, such as involuntary incidents or cyberattacks.

Finally, the underground mine is moving towards a digital mining transformation linked to the Industry 4.0 paradigm. In this sense, in practical terms, the underground mining operations of the future will require less human participation, giving greater importance to machines and robots, thus being an automated activity. This is how the integrated set of sensor technologies, IoT, artificial intelligence, digital twins, robotics, and automation will allow greater security to be provided to mining operators, reduce uncertainties/risks, and have greater control over the quality of work in the underground mine in real-time and at all times of the mining cycle. The implementation of solutions, such as IoT LoRaWAN-based WSNs will allow a step towards a wireless future and the planning of an efficient, safe, and sustainable underground mining work environment. 

## Figures and Tables

**Table 1:** Searching index of the literature search method according to the mentioned databases.

Searching Index	Content
Time	2017–2024
Database	Scopus and Web of Science
Title/Subject-Abstract	“IoT” AND “LoRa” AND “LoRaWAN” AND “sensor” AND “Underground Mine”
Publication Type	“Article” and “Review Article”
Data Extraction Index 1	Sensor IoT Name
Data Extraction Index 2	Connectivity Type
Data Extraction Index 3	Parameter/Measurement
Data Extraction Index 4	Implementation Status

**Table 2:** Summary of information about worldwide scientific articles published linked with IoT sensors and LoRaWAN wireless networks used in underground mine.

#	Publication Name/Authors	Sensor IoT Name	Connectivity Type	Parameter/ Measurement	Implementation Status
1	Development of LoRa Communication System for Effective Transmission of Data from Underground Coal Mines/ Kumar et al., 2023, [21]	Gas monitoring sensors	LoRaWAN	CH_4_, CO_2_, and CO	Underground coal mines—operation
2	Measurements and Models of 915 MHz LoRa Radio Propagation in an Underground Gold Mine/ Branch, 2022, [22]	Gas monitoring sensors	LoRaWAN	CH_4_, CO_2_, and CO	Underground gold mine—operation
3	A LoRa-Based Linear Sensor Network for Location Data in Underground Mining/ Branch and Zhao, 2020, [30]	Personnel and vehicle position	LoRaWAN	Location of mine operators and vehicles	Underground mine—operation
4	An Event Reporting and Early-Warning Safety System Based on the Internet of Things for Underground Coal Mines: A Case Study/ Jo and Khan, 2017, [31]	Sensors for control the air quality	Zigbee and Bluetooth	Temperature, humidity, CH_4_, CO_2_, and CO	Hassan Kishore coal underground mine—operation
5	Applications of the Open-Source Hardware Arduino Platform in the Mining Industry: A Review/ Kim et al., 2020, [32]	Sensors for gas, humidity, temperature, and accelerometer	GSM, Bluetooth, Wi-Fi, and ZigBee	Parameters linked to measures of atmospheric and geological information	Non-specified
6	Development of Digital Device Using ZigBee for Environmental Monitoring in Underground Mines/ Lee et al., 2022, [33]	Sensors for detecting changes in vibration and dust before and after blasting	Zigbee	Parameters linked to vibration and dust emission.	Samdo underground mine—operation
7	Three-Dimensional Model-Based Line-of-Sight Analysis for Optimal Installation of IoT Monitoring Devices in Underground Mines/ Lee et al., 2023, [34]	Sensors to monitoring the line of sight (LOS) must be connected without obstacles	Zigbee	Monitoring the parameter of line of sight (LOS) must be connected with-out obstacles	Samdo underground mine—operation
8	Deploying IIoT Systems for Long-Term Planning in Underground Mining: A Focus on the Monitoring of Explosive Atmospheres/ Medina et al., 2024, [35]	Sensors for monitoring early detection of hazardous gases and mitigation of explosion risks	Zigbee	Parameters to detect hazards gases and explosion risks	Coal underground mine—operation
9	Designing a Monitoring System to Observe the Innovative Single-Wire and Wireless Energy Transmitting Systems in Explosive Areas of Underground Mines/ Kianfar et al., 2022, [36]	Sensors to detect hazards gases and humidity	Wi-Fi	Parameters to detect hazards gases and humidity	Explosive areas of underground mines—operation
10	Sensing Technology Applications in the Mining Industry—A Systematic Review/ Duarte et al., 2022, [37]	Sensors to detect geotechnical hazards, toxics gases, and humidity	LoRaWAN, Wi-Fi, Bluetooth, Zigbee, GSM	Parameters to detect geotechnical hazards, toxics gases, and humidity	Open-pit mines and underground mines—operation
11	A Study on Industrial IoT for the Mining Industry: Synthesized Architecture and Open Research Directions/ Aziz et al., 2020, [38]	Wireless sensor network (WSN)	Zigbee	Parameters to detect geotechnical hazards, toxic gases, and humidity	Open-pit mines and underground mines—operation
12	Reliability of LoRaWAN Communications in Mining Environments: A Survey on Challenges and Design Requirements/ Musonda et al., 2024, [23]	Sensors to detect geotechnical hazards, toxic gases, and humidity	LoRaWAN, Wi-fi, Zigbee, Bluetooth, 5G	Parameters to detect geotechnical hazards, toxic gases, and humidity	Underground mine—operation
13	Communication of Sensor Data in Underground Mining Environments: An Evaluation of Wireless Signal Quality over Distance/ Ikeda et al., 2021, [25]	Strain gauge sensor installed in a narrow reef stope, horizontal tunnel, and vertical shaft area	Wi-Fi	Parameters to detect deformations and displacements in tunnels	Underground mine—operation
14	Development of a Low-Cost Device for Monitoring Ventilation Parameters (Temperature, Humidity and Pressure) in Underground Environments to Increase Operational Safety Using IoT/ Pedrosa Santos et al., 2022, [39]	Low-cost sensors to monitor temperature, humidity, and pressure	4G	Parameters for monitoring gases, such as CH_4_, NOX, NH_3_, H_2_S, SO_2_, and NOX, among others	Underground mine—operation
15	An Integrated Environment Monitoring System for Underground Coal Mines—Wireless Sensor Network Subsystem with Multi-Parameter Monitoring/ Zhang et al., 2014, [26]	Sensors for monitoring smoke, coal dust, temperature, and humidity	Zigbee	Parameters for monitoring are particle matter and gases, among others.	Underground coal mine—operation
16	An Internet of Things System for Underground Mine Air Quality Pollutant Prediction Based on Azure Machine Learning/ Jo and Khan, 2018, [40]	Sensors for monitoring air quality	Zigbee	Parameters to be monitored, with CH_4_, CO, SO_2_, and H_2_S as the most influencing gases	Underground mine—operation
17	Hybrid Blockchain and Internet-of-Things Network for Underground Structure Health Monitoring/ Jo et al., 2018, [41]	Sensors for monitoring geotechnical and environment issues	Wi-Fi	Parameters to be monitored displacement, strain, and temperature	Underground mine—operation
18	Use-Case-Oriented Evaluation of Wireless Communication Technologies for Advanced Underground Mining Operations/ Theissen et al., 2023, [42]	Sensors for monitoring geotechnical and environment issues	LoRa, Bluetooth, Wi-Fi, 5G	Parameters to be monitored, displacement, strain, and temperature	Underground mine—operation
19	Robust Localization for Underground Mining Vehicles: An Application in a Room and Pillar Mine/ Inostroza et al., 2023, [43]	Sensors for monitoring 2D LIDAR	4G	Parameters to be monitored, 2D representations/maps of the environment	Underground mine—operation
20	A New Internet of Things Hybrid VLC/RF System for m-Health in an Underground Mining Industry/ Iturralde et al., 2024, [44]	Sensors to monitor vital signals of mine operators	Zigbee, Wi-Fi, Bluetooth	Parameters to be monitored pulse rate, respiratory rate, and body temperature	Underground mine—operation
21	Real-Time Monitoring of Underground Miners’ Status Based on Mine IoT System/ Jiang et al., 2024, [45]	Sensors to monitor gases and vital signals of mine operators	Bluetooth, 5G	Parameters to be monitored, toxic gases, pulse rate, respiratory rate, and body temperature	Underground mine—operation
22	Bluetooth Beacon-Based Mine Production Management Application to Support Ore Haulage Operations in Underground Mines/ Park and Choi, 2021, [46]	Sensors to monitor ore haulage vehicles	Bluetooth	Parameters to be monitored in real-time position of vehicles	Underground mine—operation

**Table 3:** General comparison of different technologies for wireless monitoring in underground mine environments. Adapted from [21,30].

LAN		Cellular		Low Power WAN	
Short-Range Communicating Devices		Long-Range with Power with M2M		Long-Range with Battery and IoT	
Examples: Zigbee, Bluetooth, and Wi-Fi, among others.		Examples: GSM (Global System for Mobile Communications), 3G, 4G, and 5G, among others.		Examples: LoRaWAN, Sigfox, LTE, NB-IoT, and CAT-M, among others.	
Advantages: -Mobile -In-home -Short-range -Factory automation	Disadvantages: -Battery life -Long range	Advantages: -Long-range -High data-rate -Coverage	Disadvantages: -Battery life	Advantages: -Long-range -Long battery -Low-cost	Disadvantages: High data-rate

**Table 4:** Comparison, in detail, of main technologies for wireless monitoring in underground mine environments. Adapted from [22,23,37].

Attributes/IoT Technology	Zigbee	Bluetooth	Wi-Fi	GSM	LoRaWAN
System resource requirements	4 KB–32 KB	>250 KB	>1 MB	>16 MB	>16 MB
Power waste	Low	Common	Common	High	Low
Battery Life	100–1000 days	1–7 days	1–5 days	1–7 days	5–10 years
Number of nodes in local network	6500	7	30	1000	1000
Data rate	20–250 Kbps	1000 Kbps	11,000 Kbps	64–128 Kbps	0.3–50 Kbps
Communication distance	10–100 m	1–100 m	1–100 m	1000 m	10,000–20,000 m (*)
Data power	12.3 dBm	−20 to +20 dBm	23 to 26 dBm	Up to 6 W	10–30 dBm
Frequency bands	784 MHz, 868 MHz, 915 MHz, 2.4 GHz	2.4–2.485 GHz	900 MHz, 2.4 GHz, 5 GHz, 6 GHz	3.3–4.2 GHz, 24–54 GHz	470–510 MHz, 863–870 MHz, 902–928 MHz
Power consumption	10–100 mW	100 mW, 2.5 mW and 1 mW, BLE: 0.01–0.5 W	9–12 W	1000 W to 20 KW	−137 to −150 dBm
Standards	IEEE 802.15.4 [50]	IEEE 802.15.1 [51]	IEEE 802.11 [52]	IEEE 802.11 [52]	IEEE 802.15.4 [53]
Advantages	Reliability, low-cost, low power consumption	Low-cost and easy to operate	High speed and adaptability	Good transmission quality and large coverage range	Uses fewer gateways, ultra-low power consumption, adaptive rate of data transmission
Disadvantages	Suitable for indoor applications only, short-range depends on cellular coverage for off-site monitoring; prone to network interference and channel noise and not very secure	Range of up to 100 m, energy-inefficient in real-time applications and drains battery if left on, slower data transmission rate, security vulnerabilities	Relies on Ethernet as backbone; high power consumption and low capacity in terms of the number of devices supported; practically, with one access point at 2.4 GHz, the range is short, within 200–300 m	Unavailable in remote locations where mines exist, high power consumption, devices are more complex, not suitable for energy-constrained IoT devices, coverage distance is short underground	Aloha MAC protocol can cause data collisions and increase delay, requires a subscription with a single vendor, unsuitable for video surveillance due to low data rates
Applications	Wireless detection and control	Short-distance data transmission	Many data transmissions, such as web access and video	Voice and data transmission	Long-range data transmission

(*) Note: the range for LoRa performance depends on the environment; 10,000–20,000 m are obtained only in certain favorable conditions. These values could express a maximum range. Different models of Lora technology work on different frequency bands and for that the range changes. This depends on the local and regional regulations [21,23].

**Table 5:** Comparison of different typical network topology types for application in mining project environments. Adapted from [60].

Network Topology	Typical Connectivity	Sensor Failure Consequences	Critical Component	Favorite Application in Mining Facilities
Point-to-point	Hardwired	Total system failure, if one sensor fails	Connection between two points	Mining camps
Bus	Hardwired	Total system failure, if one sensor fails	Collision avoidance system to resolve issues when two nodes simultaneously send data	Mining roads and mining camps
Linear	Hardwired	Failure of those depending on that node, if one sensor fails	Node dependence to propagate the data	Metallurgical process plant
Star	Wireless	Localized to a single node, if one sensor fails	Intelligent central node	Mine tailings storage facility, road mine network, and metallurgical process plant
Tree	Wireless	Failure of those depending on that node, if one sensor fails	Processing power as the node gets further from the root node	Underground mine and open pit mine
Mesh	Wireless	Localized to a single node, if one sensor fails	Self-healing ability, redirecting data along different paths if a node fails	Underground mine and mine tailings storage facility

## Data Availability

The data presented in this study are available on request from the corresponding author.

## Abbreviations

BATs	Best Available Technologies
BAPs	Best Applicable Practices
BEPs	Best Environmental Practices
IT	Information Technology
OT	Operational Technology
ICTs	Information and Communication Technologies
AI	Artificial Intelligence
IoT	Internet of Things
IIoT	Industrial Internet of Things
DT	Digital Twins
FoS	Factor of Safety
M2M	Machine to Machine
PPEs	Personal Protection Elements
LiB	Lithium-ion Battery
WSNs	Wireless Sensor Networks
GSM	Global System for Mobile Communications
3G	Third-Generation Cellular Technology—Mobile Network that Combines Digital Voice Signals and Mobile Data
4G	Fourth-Generation Cellular Technology—Mobile Network that Supports BroadBand Mobile Data
5G	First-Generation Cellular Technology—Mobile Network that Provides More Connectivity and Faster Connection Speeds
ISM	Industrial, Scientific, and Medical
APPs	Software Applications
Wi-Fi	Wireless Fidelity
LAN	Local Area Networks
WAN	Wide-Area Network
NB-IoT	NarrowBand Internet of Things
CAT-M	Category M
LTE	Long Term Evolution
LoRa	Long Range Communication (Physical Layer)
LoRaWAN	Long Range Wide Area Network (Communication Protocol and Physical Layer)
MAC	Media Access Control (Address)
IP	Internet Protocol (Address)
KPI	Key Performance Indicator
ALARP	As Low As Reasonably Practicable
masl	Meters above sea level
